# Moderate Aerobic Exercise Enhances the Th1/Th2 Ratio in Women with Asthma

**Published:** 2019-03

**Authors:** Azam Zarneshan, Mahdia Gholamnejad

**Affiliations:** 1 Department of Exercise Physiology, Azarbaijan Shahid Madani University, Tabriz, Iran,; 2 Department of Pulmonology, Urmia University of Medical Sciences, Urmia, Iran

**Keywords:** Aerobic exercise, Asthma, IFN-γ, IL-4, Steroid hormones

## Abstract

**Background:**

In this study, we aimed to investigate the effects of aerobic exercise training on the serum IL-4/IFN-γ ratio (Th1/Th2 balance), testosterone/cortisol ratio, levels of cortisol, testosterone, estrogen, and progesterone, and body mass index (BMI) and to determine the relationship between changes in these variables in women with asthma.

**Materials and Methods:**

Twenty-one women with mild to moderate asthma and regular menstrual cycles were selected in this study. Eleven women in the exercise group participated in the aerobic exercise program (60 min/day, three days a week in the evening). Peripheral blood samples were collected before (week 0) and after (week 12) the program. The samples were analyzed to determine the levels of sex hormones and cortisol via chemiluminescence assay, and cytokines were examined by ELISA assay.

**Results:**

The findings showed a significant increase in the Th1/Th2 ratio and a decrease in cortisol and BMI in the exercise group, compared to the control group (P<0.05). There was no significant correlation between changes in cortisol, sex hormones, and BMI and the increase in Th1/Th2 ratio.

**Conclusion:**

The present results suggested that moderate aerobic exercise enhances the Th1/Th2 ratio, independent of changes in steroid hormone level and BMI in women with asthma.

## INTRODUCTION

Asthma is a complex, heterogeneous, and chronic inflammatory disease of the airways, which affects nearly 300 million people worldwide (1). Chronic inflammation of the airways, caused by cytokines and other inflammatory mediators (2), is still a matter of controversy among researchers. Evidence suggests that Th2 cytokines (IL-4, IL-13, and IL-5) play pathogenic roles in allergy and asthma (3, 4). Additionally, Th1 cells, through production of interferon-gamma (IFN-γ), can suppress the Th2 effector functions in the development of asthma (5, 6). It has become increasingly clear that regulation of Th1/Th2 balance is a key contributor to asthma immunotherapy (7–9).

There is no available treatment for asthma, and all treatment guidelines only emphasize on the clinical control of this disease (7). Therefore, use of different preventive methods, such as active lifestyle, may be effective. In the past two decades, several studies have examined the effect of exercise intensity as a non-medical factor in reducing inflammation and intensity of asthma. In this regard, some studies have reported the positive effects of aerobic exercise training on reducing the inflammatory markers (10–12). A study by Cordova-Rivera et al. showed that higher levels of physical activity were associated with lower levels of systemic inflammation in patients with severe asthma (10). Moreover, De Araujo et al. revealed that regular moderate aerobic training could prevent and reduce lung inflammation by increasing the level of Th1 and decreasing Th2 (11). Also, aerobic exercise has been shown to decrease Th2 response and airway inflammation in a murine model of asthma (12).

Sex is one of the various regulatory factors, which controls the Th1/Th2 balance (13). There is a growing body of research on the role of sex hormones in immune cell and T-cell regulation and development of asthma in women (14, 15). Accumulating evidence suggests that sex hormones modify the Th1/Th2 balance by increasing Th2 immune responses in women (15, 16). It has been reported that progesterone and estrogen may have synergistic effects on the Th1/Th2 balance and exacerbation of asthma in women. Androgens, on the other hand, shift the Th1/Th2 balance towards a Th1 phenotype (16). According to previous studies, testosterone is likely to restrict immunological and inflammatory processes exacerbating asthma, which may be one of the reasons for the low prevalence of asthma in men, compared to women (17).

Although testosterone is produced in small quantities in the ovaries and adrenal glands, it is an essential hormone for women (18), associated with asthma (19). On the other hand, according to recent findings, cortisol induces a shift in the Th1/Th2 balance towards Th2, compared to testosterone. It inhibits the production of Th1 cytokines and up-regulates the production of Th2 cytokines by immune cells (20, 21). The role of glucocorticoids in Th1/Th2 patterns (21), as well as the relationship between psychological stress, cortisol level, and asthma, has been examined in the literature (22).

In addition to sex hormones and cortisol, obesity also affects the Th1/Th2 immune imbalance (23, 24) and is considered a risk factor for the development of asthma (25). The association of obesity with the risk of asthma is higher in women than men (26). On the other hand, in obese asthmatic patients, aerobic exercise training may reduce obesity and change the levels of cytokines, which are associated with obesity and asthma. Physical activity also changes the level of female sex hormones (27) and cortisol (28). Therefore, alterations in steroid hormones may be associated with changes in the Th1/Th2 ratio due to exercise.

One of the therapeutic methods for asthma is to restore the Th1/Th2 balance (9). To answer the question of whether changes in obesity and steroid hormones due to exercise training play a role in improving of the Th1/Th2 balance in women, the current study aimed to investigate the effect of 12 weeks of aerobic exercise on changes of Th1 (IFN-γ) and Th2 (IL-4) cytokines, as well as effective factors, including cortisol, testosterone, estrogen, progesterone, testosterone/cortisol ratio, and body mass index (BMI) in asthmatic women.

## MATERIALS AND METHODS

### Subjects

In this clinical trial study, twenty-one inactive women with mild to moderate asthma and regular menstrual cycles (mean age: 35.63±7.4 years; range: 25–42 years) were recruited from Sahand Asthma Clinic in Urmia, Iran. The participants were non-smokers and did not use any hormonal drugs. They also had no major cardiovascular, renal, metabolic, or pulmonary diseases and had a history of regular menstrual cycle over the past three months. Also, they had not participated in regular exercise or diet programs over the past six months. Before the exercise program, a written informed consent was obtained from each participant. All aspects of the study involving human subjects were approved by the local medical ethics committee. Also, all study procedures were in accordance with the principles of the Declaration of Helsinki.

The participants were divided into two groups: exercise (n=11) and control (n=10). The control group received routine medical treatment and did not partake any exercise sessions; their only physical activity involved work or household chores. On the other hand, the exercise group, besides routine medical care, participated in a specific exercise program for 12 weeks (three sessions of aerobic exercise per week in the evening).

### Menstrual cycle control

We monitored the menstrual cycle of all participants for at least three months before the intervention. Subjects with irregular menstrual cycles were excluded from the analyses. After recording the length of menstrual cycle, day of ovulation was determined based on the instructions of MAX14 kit (an ovulation predictor kit) and body temperature control. Six to eight days after ovulation (almost day 21 of a 28-day cycle) was considered as the luteal phase. All measurements and blood collections were performed in a constant menstrual phase (mid-luteal phase) in pre- and post-exercise periods.

### Training protocol

The exercise program included 12 weeks of aerobic exercise for 60 minutes, three sessions a week, with an intensity of 60–80% of maximum heart rate (MHR). Each session consisted of 15 minutes of warm-up, 30 minutes of walking on a treadmill (60–80% of MHR), and 15 minutes of breathing exercises. The treadmill exercise intensity was 60% of MHR for the first two weeks, which gradually increased in the subsequent sessions by increasing the treadmill slope and speed. The intensity of exercises was controlled by a Polar heart rate monitor. To protect the patients and reduce problems during exercise, such as exercise-induced asthma, the American College of Sports Medicine (ACSM) guidelines were applied. Exercise training involved the nasal route of breathing. The patients were asked to breathe through their noses as much as possible when exercising and drink enough water before and during exercise.

### Data collection

Overnight fasting blood samples were collected, and anthropometric indices were evaluated before the first training session and 40 hours after training. The post-exercise visit took place at the same time of the day in the same phase of the menstrual cycle to minimize the effects of circadian rhythm and sex hormones on the sample content. Venous blood samples (10 mL in each visit) were collected at 8 a.m. in the luteal phase; they were allowed to settle for 20 minutes for clotting and serum separation. The serum was then collected via centrifugation. Next, 5 mL of the serum was collected to measure the level of steroid hormones, and the remaining serum was frozen until cytokine (IL-4 and IFN-γ) assays.

The level of sex hormones (estrogen, progesterone, and testosterone) and cortisol was measured by chemiluminescence assay (Liaison Diasorin, Germany). ELISA assay was also performed for the detection of cytokines, such as IL-4 and IFN-γ. The ELISA kits were purchased from Eastbiopharm (USA), involving sandwich-type assays. Bodyweight and height were measured with standard Medical Scales (Seca 755, Germany), respectively before (week 0) and after (week 12) the program. BMI was calculated by dividing the person’s weight in kilograms by the square of height in meters (kg/m ^2^). The time and dosage of medications were fixed for every subject in the pre- and post-exercise stages.

This study was approved by the Ethics Committee of Urmia University of Medical Sciences (ir.umsu.rec.1395.81) and registered in the Iranian Registry of Clinical Trials (IRCT2016052328028N1).

### Statistical analysis

Distribution of data in the exercise and control groups was evaluated using the Jarque-Bera test. Baseline characteristics were compared between the control and exercise groups, using unpaired t-test. Also, differences between the exercise and control groups were compared by ANCOVA test, with the baseline value used as a covariate. Pearson’s correlation coefficient test and stepwise multiple linear regression were used to determine the relationship between changes in the Th1/Th2 ratio, steroid hormones, and BMI in the exercise group. SPSS version 23 was used for all statistical analyses. Data are shown as mean±SD. P-value less than 0.05 was considered significant.

## RESULTS

### Subjects

The mean age of subjects with a history of asthma (mean duration: 9.5±3.9 years) was 35.6±7.4 years. There was no significant difference between the groups in terms of age, asthma duration, BMI, or serum concentration of hormones before the exercise program (P>0.05).

### Th1/Th2 ratio

There were significant differences between the groups regarding the changes in the mean IL-4 level (F(1, 18)=6.16; P=0.023) and IFN-γ/IL-4 ratio (F(1, 18)=5.88; P=0.026). We observed a significant decrease in the serum level of IL-4 and an increase in IFN-γ/IL-4 ratio in the exercise group, compared to the controls (P<0.05). However, no significant difference was observed in the serum level of IFN-γ between the groups (P>0.05) (Table 1).

**Table 1.** Changes in systemicTh1, Th2 cytokines, steroid hormones and BMI at baseline and after 12 weeks exercise intervention

**variable**	**Exercise**		**Control**		**F**	p-value^*^

	**pre**	**post**	**pre**	**Post**		
Th1/Th2(IFN-γ/ IL-4)	2/09±0/56	2/88±1/41	2/39±0/68	2/27±0/82	5/88	P<0/05 ^*^
IFN-γ(pg/ml)	30/6±8/03	32/4±8/45	28/9±4/22	29/0±4/91	0/770	
IL-4(pg/ml)	14/9±3/19	12/6±3/93	13/0±4/78	13/9±3/96	6/16	P<0/05 ^*^
Cortisol (μg/dl)	11/9±5/56	8/05±3/65	11/4±4/41	11/3±5/25	4/59	P<0/05 ^*^
Testosterone(ng/ml)	0/277±0/05	0/366±0/16	0/275±0/06	0/308±0/09	1/05	
Estrogen(pg/ml)	120/6±80/7	105/9±85/9	117/9±49/9	116/3±27/8	0/303	
Progesterone(ng/ml)	10/9±4/61	8/20±2/78	9/9±4/29	8/9±2/39	0/743	
Ts/Co ratio ^≠^	0/032±0/03	0/075±0/09	0/043±0/06	0/033±0/02	3/62	
BMI(kg/m^2^)	29/7±5/19	29/1±4/94	28/9±3/66	29/3±3/52	11/86	P<0/01^*^

* ANCOVA with baseline value as the covariate (exercise versus control).

≠ testosterone/cortisol ratio.

### Serum level of sex hormones, cortisol, and BMI

The serum level of cortisol (F(1, 18)=4.59; P=0.047) and BMI (F(1, 18)=11.86; P=0.003) significantly decreased in the exercise group, compared to the control group after 12 weeks of exercise training. However, the serum level of sex hormones (estrogen, progesterone, and testosterone) and testosterone/cortisol ratio did not significantly change in the exercise group (P>0.05) (Table 1).

### Relationship between changes in the Th1/Th2 ratio and steroid hormones and BMI

Table 2 presents the results of Pearson’s correlation test between changes in the Th1/Th2 ratio (ΔTh1/Th2) and control variables after 12 weeks of aerobic exercise training. There was no significant correlation between the level of steroid hormones and Th1/Th2 changes from the pre-exercise stage to the post-exercise stage (P>0.05). Also, changes in BMI were not significantly associated with changes in the Th1/Th2 ratio (P>0.05). However, there was a positive correlation between the baseline BMI, cortisol level, and Th1/Th2 ratio changes in the exercise group (P<0.05) (Table 3).

**Table 2.** The relationship among changes in Th1/Th2 ratio and Intervening variables in exercise group after 12 weeks of aerobic exercise training

**variable**	**Δ Th1/Th2**	

	Correlation	Sig
Δ Testosterone	0/526	
Δ Cortisol	0/347	
Δ Estrogen	−0/561	
Δ Progesterone	−0/554	
Δ Ts/Co ratio	−0/185	
Δ BMI	−0/524	
Δ Il-4	−0/689	P<0/02 *
Δ IFN-γ	0/675	P<0/05*

* Significance of change at the level of 0.05.

**Table 3.** The relationship among Th1/Th2 changes and the baseline levels of Intervening variables in exercise group

**variable**	**Δ Th1/Th2**	

	Correlation	Sig
Testosterone	−0/249	
Cortisol	−0/612	P<0/05 *
Estrogen	0/226	
Progesterone	0/330	
Ts/Co ratio	0/467	
BMI	0/619	P<0/05 *
Il-4	−0/441	
IFN-γ	−0/154	

* Significance of change at the level of 0.05.

IFN-γ (r=0.675; P<0.05) and IL-4 (r=−0.689; P<0.02) changes showed significant correlations with Th1/Th2 ratio changes (Table 2). According to stepwise multiple linear regression, only reduction of IL-4 level had significant effects on the increase of Th1/Th2 ratio after 12 weeks of training. In other words, this indicates that the IL-4 changes in regression model explain 42% of Th1/TH2 ratio changes (beta=0.689; adjusted R-squared=0.42; P<0.02).

## DISCUSSION

Today, more attention is being paid to the regulation of Th1/Th2 balance in asthmatic patients (9, 29). The present study was performed to investigate the effect of 12 weeks of aerobic exercise training on the Th1/Th2 balance in asthmatic women. Our results showed that 12 weeks of moderate aerobic training significantly increased the Th1/Th2 ratio due to the reduction of IL-4 level in the serum (Table 1).

Overproduction of IL-4 seems to contribute to the pathophysiology of asthma (30) and appears to play an important role in airway remodeling, Th2 cell development, IgE synthesis, and B-cell growth and proliferation (5, 31). In line with the findings of the present study, Vieira et al. concluded that moderate exercise training decreases the number of peribronchial inflammatory cells, expressing IL-4 in a murine model of asthma (32). Similarly, Mackenzie et al. (33) and Araújo et al. (11) reported the reduction of IL-4 level in the bronchoalveolar lavage of trained animals.

There are very few studies investigating the effect of aerobic exercise on the serum level of IL-4 in asthmatic patients (34). Fu and Yu reported that after six months of aerobic exercise, the serum level of IL-4 significantly decreased in patients with allergic rhinitis (35). Conversely, Boyd et al. showed that 12 weeks of aerobic training (40 minutes per session) could not change the serum level of IL-4 in adults with mild to moderate asthma (34). Also, Andrade et al. showed that six weeks of aerobic training did not change the serum level of IL-4 in asthmatic children (36). The higher volume, duration, and intensity of exercise training may contribute to the significant decline in the level of IL-4, compared to the exercise programs proposed by Boyd et al.(34) and Andrade and colleagues (36).

Moreover, there are controversies regarding the effects of exercise training on the level of IFN-γ. Some studies have shown that aerobic exercise does not change the level of IFN-γ (32, 36), while some have shown an increase in the level of IFN-γ (37) or a decrease in the level of IFN-γ (38). The results of the present study indicated an insignificant increase in the serum level of IFN-γ in the exercise group after the exercise program. The effects of exercise training on inflammatory mediators in asthma patients are inconclusive and heterogeneous, and there is no definite conclusion. The discrepancy between the results could be attributed to differences in the type, intensity, and duration of exercises or differences among samples (e.g., obese/non-obese, human/animal, patient/healthy, or low/high asthma severity). The time of sample collection, seasonal variations in allergen exposure, menstrual cycle, catecholamines (39), and mental stress (40) can be also effective.

Following the reduction in the IL-4 level and the insignificant increase in the IFN-γ level, we observed a significant increase in the Th1/Th2 ratio. Generally, few studies have examined the effects of moderate aerobic exercise training on the serum INF-γ/IL-4 ratio (Th1/Th2 balance) in asthma patients (11). Our results indicated that steroid hormones and BMI changes were not correlated with alterations in the Th1/Th2 ratio in the exercise group (Table 2). Sex hormones did not change in the exercise group. Evidence suggests that changes in the level of sex hormones by exercise training may be difficult and require intensive or prolonged exercises (27, 41). Besides, reduction of sex hormones has been reported in young women following high-intensity (80–85% of VO _2_ max) (42) and high-volume (five times a week for 16 weeks) (43) aerobic training. Since intensive exercises are difficult for asthmatic patients, use of long-term or high-volume exercise is suggested in future studies. It seems that discussion about the changes in sex hormones of asthmatic women requires extensive investigations.

In the present study, the serum level of cortisol and BMI showed a significant reduction in the exercise group, compared to the control group (P<0.05). Controversial findings have been reported regarding the role of cortisol level in asthmatic patients. It has been revealed that cortisol diminishes inflammation in the airways (40, 44). There is also evidence that exposure to stress and high doses of cortisol can result in immune imbalance and increase of Th2 cytokine response (40, 45). Chen and Miller evaluated the negative effects of stress and cortisol on inflammation and asthma and found that prolonged exposure to high levels of cortisol decreased the sensitivity of immune cells to glucocorticoid signaling and increased their resistance to the potential anti-inflammatory properties of cortisol; therefore, stress is harmful for asthma patients due to the increase in the hypothalamic pituitary adrenal (HPA) activity (40).

Exercise is a model of stress that affects the psychoneuroimmune and endocrine pathways (46). Evidence suggests that physical activity is related to the reduced risk of HPA axis dysregulation and cortisol secretion (47). Some findings show that regular aerobic exercise changes the HPA axis and modulates stress reactivity (48). Also, cortisol response to psychosocial stress is lower in active people, compared to the inactive ones (49). Therefore, exercise training may be useful for asthmatic patients who are refractory to glucocorticoids. Due to the significant reduction of cortisol and the insignificant increase in testosterone level, the testosteroneto-cortisol ratio (anabolic/catabolic ratio) did not change, which may suggest that our exercise training type and intensity (60–80% of MHR) were suitable for maintaining the homeostatic balance and health of asthmatic patients.

We found that the baseline cortisol and BMI had significant relationships with the Th1/Th2 ratio changes in the exercise group (Table 3). In women with a high baseline BMI, the increase of Th1/Th2 ratio was higher, and the high baseline level of cortisol diminished the increase in the Th1/Th2 ratio. This can partly reflect the beneficial effects of exercise training on the improvement of Th1/Th2 balance in obese women with low cortisol levels.

Finally, only changes in IFN-γ and IL-4 levels had significant relationships with the Th1/Th2 ratio, and multiple stepwise linear regression analysis showed that reduction of IL-4 level significantly influenced the Th1/Th2 increase (adjusted R-squared=0.42; P<0.02). Since no similar studies were found regarding the relationship of steroid hormones and BMI with Th1/Th2 ratio changes before and after exercise, we could not compare the results of this study with other research. Therefore, further studies are essential in this area.

## CONCLUSION

In conclusion, the results of the present study revealed that 12 weeks of aerobic exercise training cause a shift of the Th1/Th2 balance by cytokine changes and independent of sex hormones, cortisol, and BMI changes.
